# Human Matching Behavior in Social Networks: An Algorithmic Perspective

**DOI:** 10.1371/journal.pone.0041900

**Published:** 2012-08-22

**Authors:** Lorenzo Coviello, Massimo Franceschetti, Mathew D. McCubbins, Ramamohan Paturi, Andrea Vattani

**Affiliations:** 1 Department of Electrical and Computer Engineering, University of California San Diego, La Jolla, California, United States of America; 2 Department of Political Science, University of Southern California, Los Angeles, California, United States of America; 3 Department of Computer Science and Engineering, University of California San Diego, La Jolla, California, United States of America; Cinvestav-Merida, Mexico

## Abstract

We argue that algorithmic modeling is a powerful approach to understanding the collective dynamics of human behavior. We consider the task of *pairing up* individuals connected over a network, according to the following model: each individual is able to propose to *match* with and accept a proposal from a neighbor in the network; if a matched individual proposes to another neighbor or accepts another proposal, the current match will be broken; individuals can only observe whether their neighbors are currently matched but have no knowledge of the network topology or the status of other individuals; and all individuals have the common goal of maximizing the total number of matches. By examining the experimental data, we identify a behavioral principle called *prudence*, develop an algorithmic model, analyze its properties mathematically and by simulations, and validate the model with human subject experiments for various network sizes and topologies. Our results include i) a 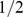-*approximate maximum* matching is obtained in logarithmic time in the network size for bounded degree networks; ii) for any constant 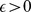, a 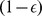-*approximate maximum* matching is obtained in polynomial time, while obtaining a *maximum* matching can require an exponential time; and iii) convergence to a maximum matching is slower on preferential attachment networks than on small-world networks. These results allow us to predict that while humans can find a “good quality” matching quickly, they may be unable to find a maximum matching in feasible time. We show that the human subjects largely abide by *prudence*, and their collective behavior is closely tracked by the above predictions.

## Introduction

The modeling and prediction of collective human behavior has been one of the key challenges of social sciences for several decades. As early as 1947, Herbert Simon argued that *information processing* constitutes the core of human decision-making [1]. A corollary of his argument is that human decision-making processes can be modeled *algorithmically*. However, such algorithmic modeling and prediction is challenging, considering that collective decision-making processes are driven by both individual attitudes and collective dynamics, and often involve social interchange and mutual agreement.

This paper argues that despite the inherent complexity of human social interactions, it is possible to isolate basic behavioral principles, formulate mathematical models, and predict collective dynamics, using an algorithmic approach. As a simple example of this approach, in the context of a distributed coordination game on networks (i.e., the *maximum matching game*), we present an algorithmic model of human behavior that is based on *simple* principles of local interaction and that is able to capture *complex* collective coordination.

Our approach is similar in spirit to the one in physics where particle systems and cellular automata described by simple rules are known to generate complex behaviors, such as phase transitions and universal computability [2]–[5]. However, our algorithmic modeling approach embeds individual interaction behavior as part of a distributed computing system and leads to computational complexity analysis.

Our work is influenced by the work of Kearns et al. [6] who studied the effect of network topology on subjects' ability to color a graph, and by subsequent work in the context of distributed coloring and consensus games [7]–[10]. However, our focus is on algorithmic modeling and analysis, rather than on observing the effect of network topology on performance.

**Figure 1:**
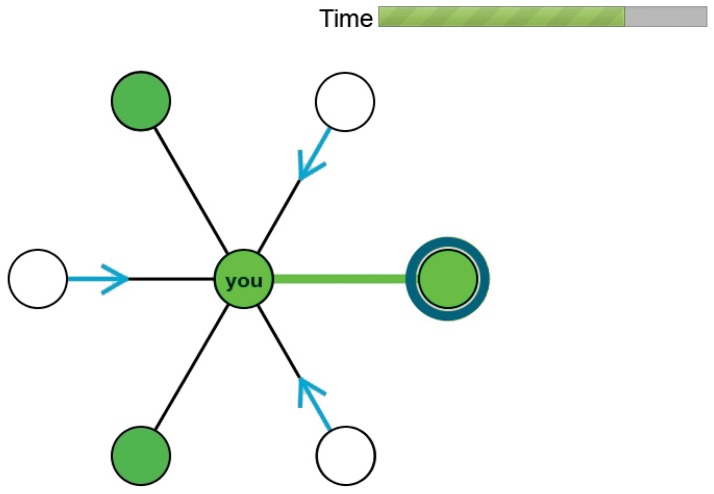
Computer interface. The subject is matched with the node on the right and is being requested by three unmatched nodes.

We have conducted over 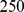 experiments with human subjects on a pool of over 80 networks with up to 24 nodes each, ranging from simple networks to more complex stochastic models including preferential attachment [11], [12] and small-world networks [13]. Our experimental set-up is simple. Subjects are represented by nodes of a network with edges representing potential matches. In our experiments, human subjects are connected over a virtual network and interact with their neighbors through a computer interface, see Figure 1. Subjects can form and destroy pairs with their neighbors, and each subject can be part of a single pair at a time. Subjects are given only local information about their immediate neighbors, and can only interact with them. They are able to propose to match with a neighbor and accept a proposal from a neighbor. While matched, a subject can also make a proposal to or accept a proposal from another neighbor; in both cases, the existing match would automatically be broken. Moreover, a subject can only have a single outstanding proposal at a time. Therefore, at any time, a subject can either be part of a matched pair, or not be matched and have at most a single outstanding proposal. Subjects are equally incentivized to achieve a *maximum matching*, namely to form the maximum number of disjoint mutual pairs, without regard to whom is matched with whom. Specifically, they are given an equal monetary reward for each game where a maximum matching is found within the allotted time.

To better understand this setup, consider the following metaphor: imagine that incoming graduate students are pairing up with faculty members. Further imagine that every member of the department prefers every graduate student to have one adviser and every adviser to have one graduate student, and only certain faculty and graduate students share interests. Communication is limited so that individuals can only tell if members with whom they share an interest are already matched. Each member of the department is now a node, the edges represent shared interest, and individuals can then propose to work with members with whom they share an edge.

Our algorithmic model is based on a simple property that we call “prudence” and that emerges from the analysis of a first set of experimental data. This property states that *individuals do not break existing matched pairs unless they receive an alternative proposal by an unmatched neighbor*. Based on this property, we propose a simple distributed algorithm, analyze its performance, validate the model with additional experimental results, and predict outcomes. The prudence property is reminiscent of the notion of risk aversion, a relevant topic in the economics literature [14], [15].

**Figure 2:**
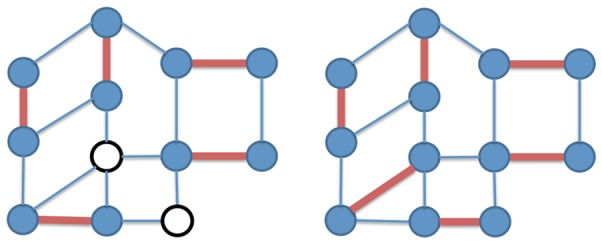
Approximate and maximum matching. Left: an approximate maximum matching of size 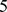 on a network with 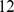 nodes (matching edges are represented in bold red, matched nodes are colored, unmatched nodes are white). Right: a maximum matching of size 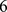 on the same network (note that the maximum matching is also a perfect matching, as all nodes are matched).

**Figure 3:**
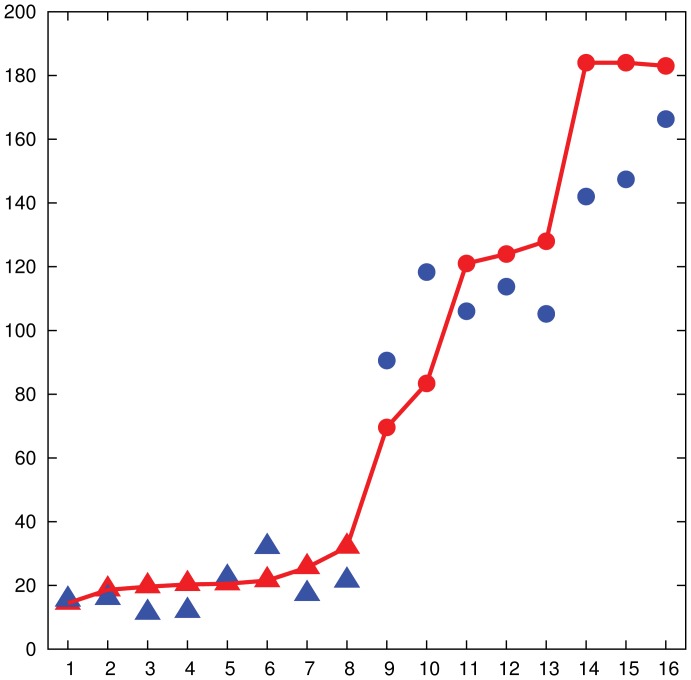
Affinity between humans' and algorithm's performance, 16-node networks. The performance of the human subjects (red points joined by continuous line) and of the algorithm (blue points) over eight bipartite 16-node networks (triangles) and eight non-bipartite 16-node networks (circles) are plotted. The experiment was run multiple times on each network and the average behavior is reported. The 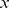-axis shows the indexes of the networks sorted by increasing average time required to reach a maximum matching. Bipartite networks are labeled from 1 to 8, while non-bipartite networks are labeled from 9 to 16. The 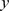-axis shows the average time (in seconds) required to reach a maximum matching for humans, while the average number of rounds of the algorithm is scaled by a constant factor.

**Figure 4:**
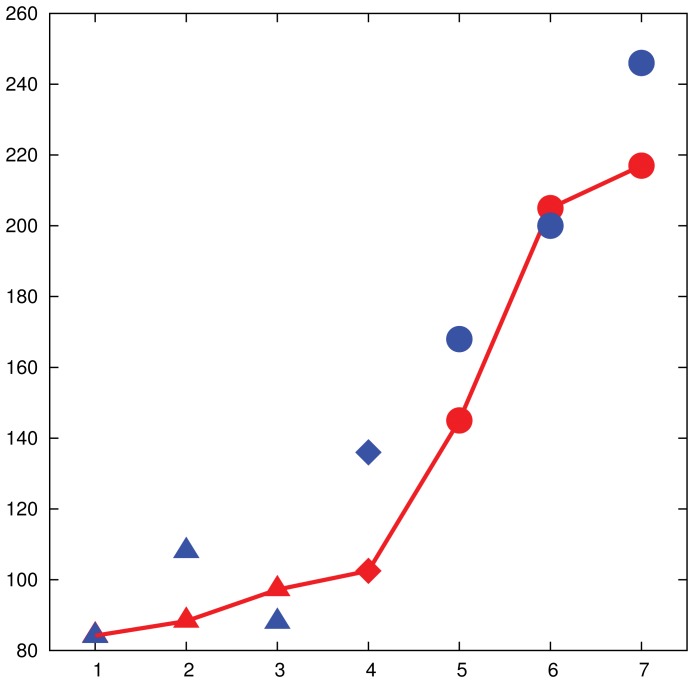
Affinity between humans' and algorithm's performance, 24-node networks. The performance of the human subjects (red points joined by continuous line) and of the algorithm (blue points) over different 24-node networks are plotted. In particular, small-world networks (triangles), a ring network (diamonds), and preferential attachment networks (circles) were tested. The experiment was run multiple times on each network and the average behavior is reported. The 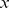-axis shows the indexes of the networks sorted by increasing average time required to reach a maximum matching. The 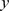-axis shows the average time (in seconds) required to reach a maximum matching for humans, while the average number of rounds of the algorithm is scaled by a constant factor.

**Figure 5:**
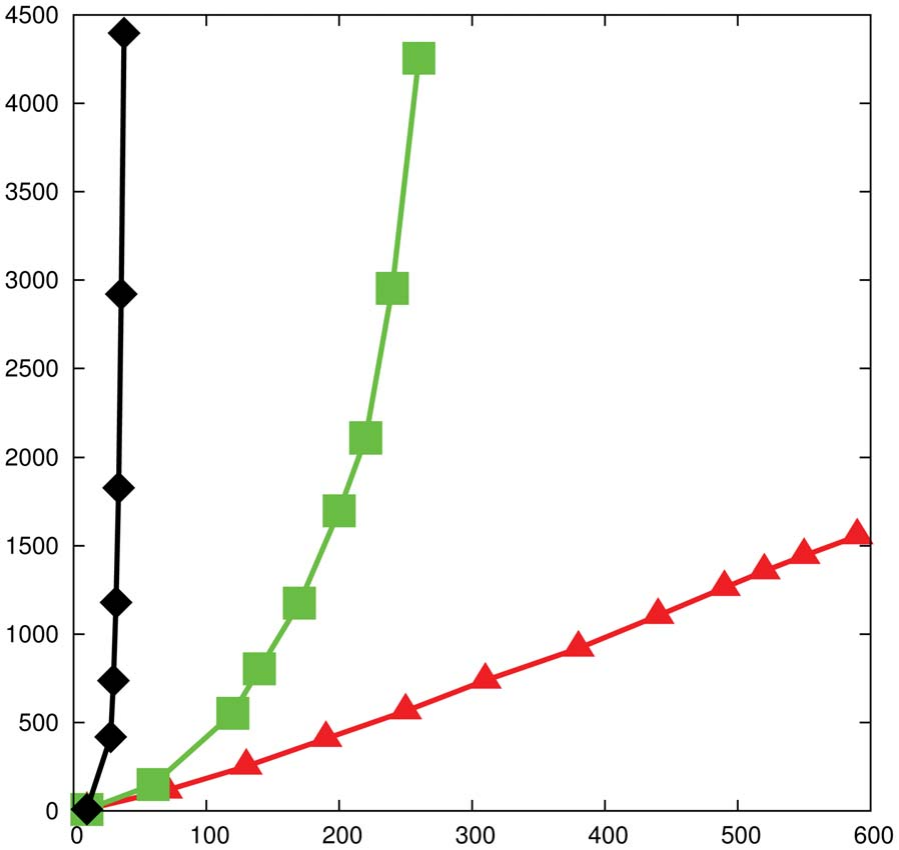
Algorithm's asymptotic performance. Prudence algorithm's performance with respect to the network's size for the “bad” graph 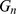 (black diamonds), for preferential attachment model (green squares), small-world model (red triangles). For each generative model and network size we generated 100 networks and run the algorithm 1000 times on each. The average behavior is reported. The 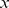-axis shows the network size, and the 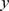-axis shows the average number of rounds required by the algorithm to converge to a maximum matching.

**Figure 6:**
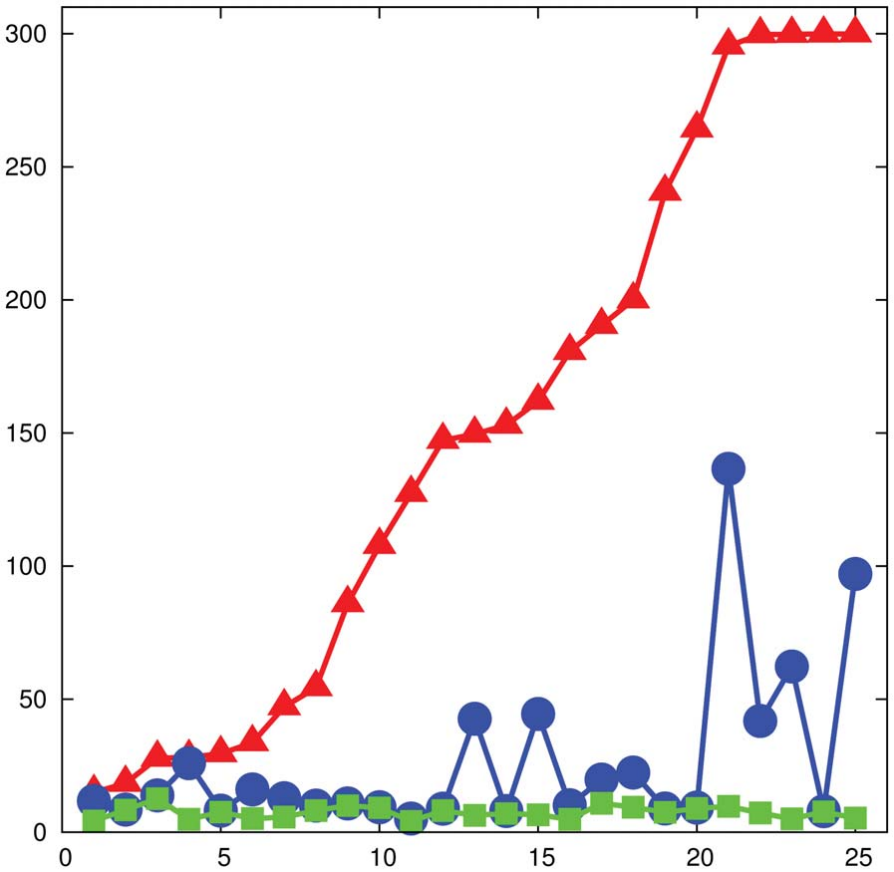
Experimental performance, 24-node networks. Performance of the experimental subjects on networks of 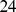 nodes. The plot shows the time to reach a perfect matching of size 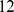 (red), an approximate matching of size 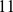 (a 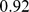
–approximate matching, in blue) and a matching of size  (a 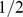
–approximate matching, in green). Results for single games are reported. The 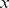-axis shows the indexes of the games sorted by increasing solving time, while the 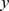-axis shows the time in seconds. The right-most four games on the red plot did not converge to a maximum matching and correspond to three instances of the “bad” graph 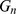 and to one instance of the preferential attachment network.

We now briefly summarize our findings. Throughout the paper we use the graph-theoretic terminology, according to which a matching is a set of edges without common nodes. The size of a matching is the number of edges in it. A maximum matching is a matching with the largest size. For 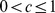, a matching is a 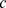-approximate maximum matching if its size is within a factor of 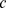 from that of a maximum matching. A matching 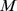 is maximal if it is not a proper subset of any other matching, i.e., for any new edge added to it, it is no longer a matching. Figure 2 depicts an approximate and a maximum matching of a network. We show that the convergence time to the maximum matching in computer simulations of the prudence algorithm fits well the experimental data (after scaling by a constant factor), see Figures 3 and 4. By computer simulations we also predict that convergence to a maximum matching is slower on preferential attachment networks than on small-world networks, see Figure 5. This prediction is validated by our experiments with human subjects. It is also in agreement with the experimental results by Kearns et al. [6] regarding the coloring problem, and with the theoretical results by Montanari and Saberi [16] regarding the spread of innovation in networks. On the theoretical side, we analyze the dynamics of the prudence algorithm and show that for all graphs of bounded degree a 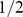-*approximate maximum* matching is reached quickly, on average in 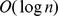 rounds, where 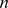 refers to the number of nodes in the network (Theorem 1); and for all graphs a 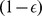-*approximate maximum* matching is reached in polynomially many rounds with high probability (Theorem 2). We also show that there are instances (called “bad” graphs) for which reaching a *maximum* matching requires exponential time with high probability when starting from a set of configurations (called “bad” matchings) which constitute almost all possible configurations (Theorems 3 and 4). These results show that in the worst case there is an exponential gap between reaching a good matching (i.e., an approximate maximum matching whose cardinality is close to a maximum matching) versus the best (i.e., perfect) matching. The experimental data shows (consistently with the theoretical analysis) that human subjects always find a “good” matching quickly, while they can take much longer to improve the solution to a maximum matching, see Figure 6. In particular, on the bad graph, human subjects could not converge to a maximum matching in the allotted time.

### Related Literature

The experimental study of human strategic behavior over networks is a topic of great current interest in the literature. The work by Kearns and others on network coloring and consensus games [6]–[10] has been particularly influential. Judd et al. [17] investigated how subjects choose between playing either a dominant or a submissive role in a network game, documenting the importance of fairness. Kearns et al. [18] performed experiments on network formation games when there is a cost for creating links. Suri and Watts [19] conducted experiments in which individuals connected over networks play local public good games. Wang et al. [20] studied multi-player prisoner's dilemma games in which subjects can propose and delete links to other players, showing that partner selection increases cooperation. Brautbar and Kearns [21] introduced a network formation game in which players need to maximize their clustering coefficients. Compared to these previous works, we focus on isolating behavioral principles of human interaction (in the context of maximum matching games) and using these principles to formulate algorithmic predictions of outcomes.

As social interaction naturally induces strategic behavior, our work is also closely related to game theory. Indeed, several authors proposed game theoretical models of human interaction over social networks. Topics vary from diffusion and contagion over networks [16], [22], [23] to strategic information retrieval [24], [25], models of segregation [26] and bargaining over networks [27], to mention a few. The main element that distinguishes our work from the game theory literature is that we focus on the algorithmic processes involved in strategic thinking and the ensuing collective dynamics rather than on equilibria. Moreover, our algorithmic model is motivated and supported by experimental data.

Finally, matching theory has received notable attention throughout the decades, both in the context of game theory and economics [28]–[31], and in the development of algorithms for the maximum matching problem [32]–[36]. We point out that our simplified setup constitutes a simplification of the richness and heterogeneity of the ties in real social networks, as the subjects have no preference over each other, all the ties are equivalent, and interaction has no cost. However, such a simplified model leads to a tractable analysis and to the formulation of a general principle of collective behavior.

## Methods

The experiments included the interaction of the participants through a computer interface, and were conducted in accordance with the ethical standards specified in the 1964 declaration of Helsinki. Written consent was granted before participation in the experiments. Our institutional review boards approved this study (UCSD IRB approval 111213SX, US Army Human Research Protection Office ARO-HRPO Log A-17038).

### The Matching Games

Before formulating our algorithmic model, we conducted four sessions of experiments, each with a different pool of sixteen undergraduate students connected over a virtual network. Subsequently, to validate our model, we ran an additional session of experiments with a pool of twenty four subjects on a set of networks that included small world and preferential attachment networks. In each of the first four sessions the subjects were asked to solve the matching game on a pool of over 70 networks. All networks admitted a *perfect matching*, namely a maximum matching with no unmatched nodes. We considered networks classified into four groups: bipartite networks admitting a unique perfect matching, bipartite networks admitting multiple perfect matchings, non-bipartite networks admitting a unique perfect matching, non-bipartite networks admitting multiple perfect matchings. Within each group, networks were randomly generated. As a remark, a bipartite network is a network whose nodes can be divided into two disjoint sets 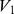 and 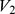 such that every edge connects a vertex in 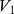 to one in 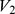. If this property does not hold, we say that the network is non-bipartite. Subjects sat in front of workstations for the entire two-hour duration of the session and had no eye-contact with each other. For each matching game, a network was chosen, subjects were randomly assigned to its nodes, and each subject interacted with its neighbors by making or accepting proposals to form matched pairs using the interface shown in Figure 1. Each subject could control the node in the center of the screen and could only see its neighbors and, among those, distinguish which of them were currently matched (marked in dark green). A subject could make proposals or accept proposals by selecting a neighbor with a mouse click, and could only have one outstanding proposal at a time to form a matched pair (circled in yellow). While subjects knew whether a neighbor is matched or unmatched, they did not have direct knowledge of any outstanding requests to their neighbors other than their own. If two neighbors selected each other, a pair was formed (a bright green link appeared between them) which could be broken when one of the partners selected another neighbor. As a remark, since a pair was formed when two subjects selected each other and each subject could make a single selection at a time, each subject could be part of a single pair at a time (with one of its neighbors).

If a perfect matching was found within the time limit of five minutes, the game was declared solved and each participant was rewarded by $.50 or $1 depending on the session, otherwise the game ended with no reward. The number of games in an experimental session was not fixed, but games were run for the two-hour duration of the session. Therefore, the number of games and the cumulative reward in a session depended on the performance of the participants, providing an additional incentive to coordinate.

The networks used in this first set of experiments can be divided into four classes: bipartite, non bipartite, unique perfect matching, multiple perfect matchings. Two one-tailed Welch's t-tests confirmed the hypotheses that it is harder for humans to complete the matching game on non-bipartite than on bipartite networks (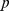-value 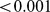); and that non-bipartite networks with unique perfect matching are more difficult to solve than non-bipartite networks with multiple perfect matchings (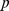-value 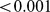). No statistically significant difference was found between the completion time of bipartite networks with unique and with multiple perfect matchings. We believe that this depended on the small network size of sixteen nodes and we did not explore larger bipartite networks further.

### The Algorithmic Model

One of the main behavioral properties that emerged from the experimental data is that matched players *may* break their current matching *only if* they have been requested by an unmatched neighbor. In particular, in 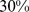 of the games no player ever violated this rule at any time during the game. In the remaining games, over 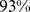 percent of the moves were in agreement with this rule. Therefore, this property led to the following modeling assumption:

**Assumption 1 (Prudence)** *A matched node does not break its current matched pair if it does not receive any request from other neighbors.*

Two remarks are in order. First, note that this behavioral rule is peculiar to the matching problem since each player needs to choose a partner but also needs to be chosen. Second, notice that a matched subject with unmatched neighbors has some incentive to behave non-prudently and break the current match, because the subject can infer from the status of its neighbors that the perfect matching is not reached yet. However, experimental data shows that this rarely happens.

For each node 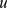, let 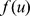 indicate 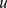
's current preference. In other words, 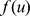 is the unique node to which 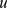 has currently proposed to. 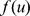 will be null if 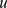 does not have a current proposal. If two neighbors 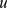 and 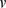 currently prefer each other (i.e., 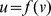 and 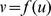), then consider them matched and the edge 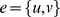 as part of the matching. Assume that each node knows if a neighbor is matched or unmatched.

Given the prudence property, we model the algorithmic behavior of humans using the 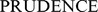 algorithm shown in Table 1. The algorithm is specified by the implementation of two functions, called 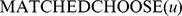 and 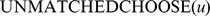, which are placeholders for the behavior that node 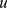 would follow depending on whether 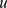 is matched or unmatched. We consider a synchronous setting, in which time is divided into rounds, and at the beginning of each round each node observes its status and the status of its neighborhood and then decides on an action to take.

**Table 1:** The algorithm.

**if** unmatched
Set 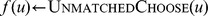
**else if** matched **and** 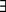 neighbor 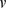 s.t. 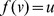
Set 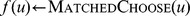
**end**

Prudence algorithm for node 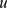.

In the following we provide a canonical implementation of the functions 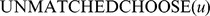 and 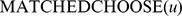 consistent with the prudence property. 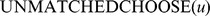 does not change the current value of 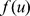 with probability 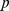, while with probability 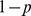 accepts the proposal from a neighbor uniformly at random from among the neighbors 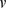 with 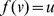 if any; if there is no neighbor 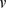 with 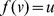, then it proposes to a node uniformly at random from among the unmatched neighbors if any; otherwise it proposes to a node uniformly at random from among all the matched neighbors. In other words, unmatched nodes prefer neighbors who requested them over other unmatched neighbors, and unmatched neighbors over matched neighbors. As for matched nodes, 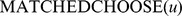 accepts a proposal from a neighbor uniformly at random from among the neighbors 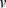 with 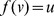 (note that 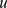
's current partner is one of them). We remark that the simulations' performance and the fit with the experimental data was practically insensitive to the value of 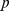 chosen in the run of the algorithm.

## Results

### Mathematical Results

In this section we present our analytical results regarding the convergence behavior of the Prudence algorithm. In particular, our results describe how well the algorithm performs in finding a large matching and the time it takes in terms of the number of rounds required. Due to space constraints, we only present proof sketches here. Complete details of the proofs are deferred to the SI.

We define a *matching* at round 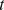 as the set of matched edges at the beginning of round 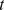 of the algorithm. We first claim that the prudence property implies that the size of the matching does not decrease with time. The proof is immediate and it is omitted.

**Claim 1** *The size of the matching at round* 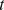 *is non-decreasing as* 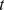 *increases.*

We then observe that the behavior of the Prudence algorithm can be described by a Markov chain over matchings. A transition from a matching 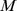 to a matching 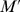 is made by selecting an edge 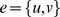 such that at least one among 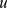 and 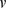 is unmatched, and setting 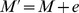 if 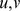 are both unmatched, and 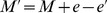 if exactly one of 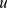 and 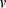 is matched in 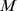 and 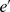 is the matching edge. This Markov chain is reversible when restricted to matchings of the same size. Since the Markov chain is memory-less and has positive probability of reaching a maximum matching, we conclude that the Prudence algorithm enjoys self-stabilization.

**Claim 2** *The* Prudence *algorithm is a self-stabilizing algorithm.*

Our first theorem says that a 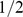-approximate matching will be reached quickly in networks with bounded degree.

**Theorem 1** *In any bounded-degree graph on* 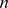 *nodes, the expected number of rounds for the* 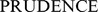 *algorithm to reach a* 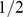 *-approximate matching is* 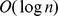.

The key idea of the proof is to show that, in expectation, the “distance” in terms of number of matched pairs to the smallest *maximal* matching shrinks by a constant factor in each round of the Prudence algorithm. Since it is well known that any maximal matching is a 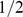-approximation of the maximum matching, the result then follows.

We remark that the assumption of having bounded degrees is necessary as there are unbounded degree graphs in which a polynomial number of rounds is required with high probability to achieve a 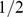-approximation. However, in this case, a polynomial number of rounds is also enough to achieve *any* constant approximation: indeed, as the next theorem states, the Prudence algorithm provides a PTAS (polynomial time approximation scheme) for the maximum matching problem. Given a graph 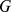, 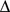 denotes its maximum degree.

**Theorem 2** *For any graph* 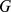 *of* 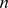 *nodes,* 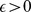 *and* 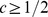, *the* Prudence *algorithm reaches a* 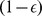 *-approximate matching in* 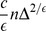 *rounds with probability at least* 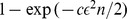.

The theorem implies that, for any constant 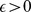, a matching whose size is within a 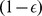 fraction of the size of the maximum matching is reached in polynomial time. For bounded-degree graphs, this result also holds for 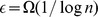, implying that in this case a maximum matching can be reached in polynomial time.

To prove the theorem, we track the progress of the algorithm towards an approximate maximum matching, using the concept of an *augmenting path*. An augmenting path is a path of odd length which alternates between matched and unmatched edges and whose extreme edges are unmatched. It turns out that there is a close connection between the size of a shortest augmenting path in a matching and how close the matching size is to the size of a maximum matching. More specifically, we use the following lemma due to Hopcroft and Karp [32].

**Lemma 1** *Consider any matching* 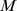 *that does not admit augmenting paths of odd length* 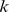 *or smaller. Then, the size of* 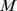 *is at least a fraction*  *of the size of a maximum matching.*

Hence, to prove Theorem 2, we need to show that short augmenting paths (for a suitably chosen 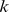) are solved in a short amount of time. It is useful to consider a particle analogy to understand the process that eliminates short augmenting paths. We consider each unmatched node as a particle. Particles move around the graph from node to node as nodes change their status between matched and unmatched states dictated by the random choices in the algorithm. There are exactly two particles along an augmenting path, situated at the extreme nodes. To understand how an augmenting path gets shorter and eventually vanishes, we consider how the two particles move closer to each other along the path.

Let 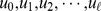 denote a *shortest* augmenting path. If the extreme unmatched node 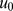 proposes to 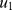 and 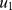 accepts the proposal breaking the current match with 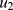, then the particle moves from 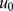 to 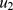. A similar argument applies to the other end of the path. Also, the minimality of the path guarantees that the internal nodes do not change their current matching as they have no unmatched neighbor. It follows that the particles become closer to each other and the augmenting path gets shorter. Using this approach, we can prove that with suitable probability the length of the *shortest* augmenting path shrinks after each round. When an augmenting path becomes an edge (that is, a path of length one), and if the extreme unmatched nodes select each other as partners, the particles and the path vanish, yielding an increment to the size of the matching. Hence, a key step of our proof is to lower bound the probability that an augmenting path of length 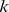 vanishes, and then to apply Lemma 1 to relate the existing augmenting paths and the matching size.

**Figure 7:**
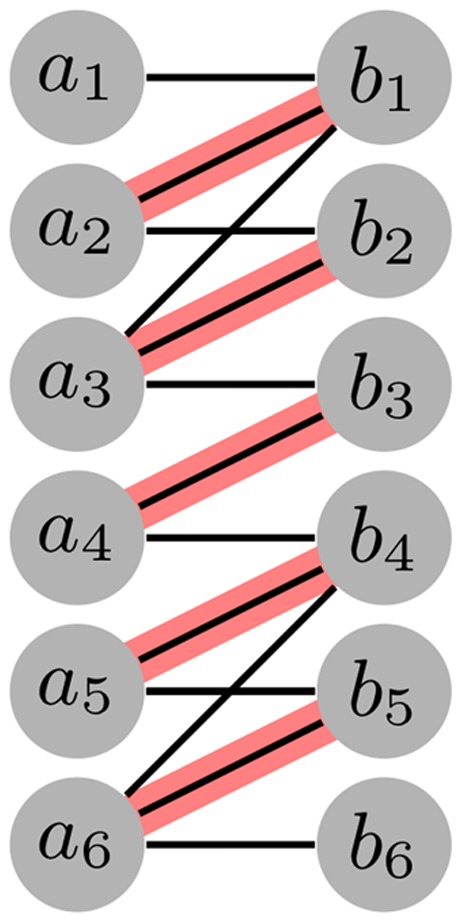
The bad graph. The “bad” graph 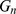 for 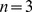. One of the “bad” matchings of Theorem 3 is highlighted in red.

We remark that the random process governing the movement of the particles in the network is not a classical random walk over the nodes of the graph. Indeed, if that were the case, a *maximum* matching would always be reached in polynomial time by a simple cat-and-mouse argument. Instead, a random move of a particle depends on the current matching, which in turn changes when the particle moves. This modest difference can lead to an exponential time gap between convergence to an approximate matching and convergence to a maximum matching. Indeed, exploiting the dependence of the particles' movements on the current matching, we show that there is a family of graphs for which the Prudence algorithm takes exponentially many rounds with high probability to reach a maximum matching starting from a set of configurations that cover almost all possible cases. This family of “bad” graphs is defined as follows (see also Figure 7).

**Definition 1 (Bad graph** *G_n_* **)** *The bipartite graph* 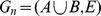 *has* 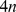 *nodes* 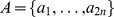 *and* 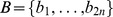, *and its edges are* 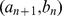, 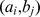 *for all* 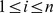 *and* 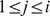, *and* 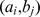 *for all* 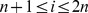 *and* 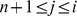.

Note that the set of “horizontal” edges 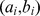, for 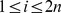 is the unique perfect matching for 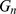.

**Theorem 3** *The* Prudence *algorithm requires* 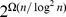 *many rounds with high probability to reach the perfect matching when starting from any* 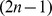 *-matching in which the two unmatched nodes are in opposite sides of* 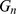.

The main idea of the proof is to track the positions of the unmatched nodes throughout the course of the algorithm and to lower bound the number of rounds needed before they meet as an adjacent pair.

We first prove a one-to-one correspondence between the Markov process of the state evolution between matchings and a classical random walk on a tree (represented in Figure S1) whose size is exponential in 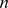. We show that this classical random walk takes exponential time to reach the root of the tree starting at any one of its nodes, thus providing a lower bound on the convergence time of the 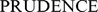 algorithm.

We say that a matching 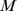 of 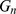 of size 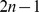 is *bad* if the 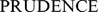 algorithm requires exponentially many rounds with high probability to converge to the perfect matching when starting from 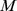. Observe that all matchings considered by Theorem 3 are bad. The following theorem states that *almost all* matchings of size 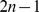 are bad.

**Theorem 4** *The ratio between the number of “bad” matchings and the number of all* 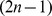 *-matchings of* 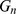 *is* 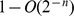.

Theorems 3 and 4 show that the 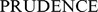 algorithm requires exponentially many rounds to converge to the perfect matching of 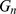 when starting from a set of configurations (the bad matchings) constituting almost all possible cases (the matchings of size 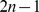).

### Validation

Figure 3 compares the performance of the human subjects (red) with that of simulations (blue) on a set of 16-node networks (8 bipartite networks and 8 non-bipartite networks) with unique perfect matchings. The networks are sorted by increasing average completion time, and as a result bipartite networks are labeled from 1 to 8, while non-bipartite networks are labeled from 9 to 16. Each of these networks was tested at least 6 times over all sessions. The vertical axis represents the time (in seconds), and the numerical values of the convergence time of the algorithm are scaled by a constant factor to best match the experimental data.

In an additional experimental session, we tested twenty four subjects connected over small-world, preferential attachment and ring networks as well as over the “bad” graph 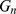. The games on the bad graph were never solved, consistent with the prediction of exponentially slow convergence. Furthermore, we found that preferential attachment networks were more difficult to solve than small-world networks (one-tailed Welch's t-test, 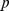-value 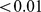). Figure 4 shows the affinity between humans' (red) and algorithm's (blue) performance, on this set of 24-node networks: small-world networks (triangles), ring network (diamonds), preferential attachment networks (circles). The 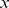-axis shows the indices of the networks sorted by increasing average time to find the perfect matching, and the 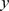-axis shows the average time.

Figure 5 shows, by simulation, that the algorithm scales linearly in the size of the network in the case of small-world networks [13], while it scales polynomially for preferential attachment networks [11], [12], and exponentially on the “bad” graph 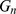. These results closely resemble the experimental data of the coloring games performed by Kearns et al. [6], where preferential attachment networks resulted in the worst performance among all tested networks, while small-worlds networks appeared to be much easier to solve.

Figure 6 shows the performance of the experimental subjects on networks of 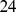 nodes, each admitting a perfect matching. In particular, it reports results for single games, and it compares the time to reach a perfect matching of size 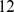 (red), an approximate matching of size 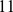 (a 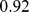-approximate matching, in blue) and a matching of size 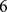 (a 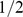-approximate matching, in green) in each game. The 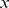-axis shows the indexes of the games sorted by increasing solution time, while the 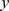-axis shows time in seconds. The plot shows (consistent with the theoretical analysis) that a 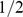-approximate matching is reached almost immediately in all games, an almost maximum matching is reached quickly, while reaching a perfect matching can take a large amount of time.

## Discussion

While it is challenging to characterize the strategies used by humans in performing even simple social tasks, as they may depend on diverse individual cognitive and psychological attitudes, we argue that it is possible to isolate simple behavioral invariants of individual behavior, which are useful for algorithmic modeling, analysis and prediction of collective dynamics of coordination.

To illustrate our approach, we have focused on a simple matching game over networks and presented a combination of theoretical, experimental, and simulation results. From the experiments, we identified the prudence property as a common behavioral invariant of human subjects when they coordinate to find a maximum matching. We proposed an algorithm as model of human behavior and showed that it can successfully predict dynamics of coordination.

We have shown that our approach is able to uncover basic behavioral properties that may not be apparent from off-line surveys. Indeed, when subjects were asked to report on their strategies in post-experimental surveys, we obtained a list of diverse strategies, including: choose a partner and never disengage from it, always accept proposals from neighbors, try to change partner if the game is not solved for a while. Moreover, our results demonstrate that algorithmic modeling and the mathematical analysis of algorithms can be useful in systematically predicting the aggregate behavior and in deriving results that hold for any graph, or for a large family of graphs. This general conclusions cannot be derived rigorously form experimental observations and computer simulations.

Our work suggests further research in several directions. A natural question is whether non-prudent behavior by a subset of the nodes can help. In a preliminary investigation, we have evaluated the performance of a variant of our algorithm where a subset of nodes behave non-prudently with a positive probability. In our simulations, these populations do not offer significant improvement in terms of finding a maximum matching. Furthermore, populations entirely composed of non-prudent nodes seem to perform poorly. In other words, a group of aggressive and risk-taking individuals might not achieve coordination easily.

Our 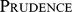 algorithm is memoryless. It is an interesting question as to what extent human subjects use memory in distributed games, and how memory could be incorporated in modeling human strategies. In an initial attempt to study this, we implemented a variant of the 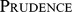 algorithm in which a node remembers its recent history and gives less preference to neighbors who recently rejected it. In simulations on preferential attachment and small world networks, memory did not result in significant improvement over the memoryless case. Furthermore, simulations show that making decisions based on events in a distant past (that is, tracking events that happened in a distant past) might hurt performance. A careful investigation of the role of memory in human strategies in distributed games is of fundamental interest.

Regarding the incentives, in our matching games each subject obtains the same reward when a maximum matching is reached, regardless of the chosen partner. How does the introduction of preferences affect the overall coordination? Preferences could be “enforced” for example by rewarding subjects based on the partners they match with. There is likely to be a trade-off between the collective task of finding a maximum matching and the individual profit maximization.

As a final remark, the proposed Prudence algorithm constitutes a *possible* reasonable explanation of human coordination behavior in the distributed matching game. Apart from the simple variations mentioned above, we did not test how well other alternative algorithmic models could fit the experimental data.

## Supporting Information

Figure S1
**Tree** 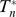 **.** Tree 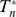 with labels, for 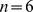.(TIF)

Text S1 **Technical proofs.** This documents contains the detailed proofs of all technical results presented in the article.
(PDF)
