# Supplementary figures and images for: Human Matching Behavior in Social Networks: An Algorithmic Perspective
Source: PLoS One. 2012 Aug 22;7(8):e41900. doi: 10.1371/journal.pone.0041900 (PMC3425504; doi:10.1371/journal.pone.0041900)

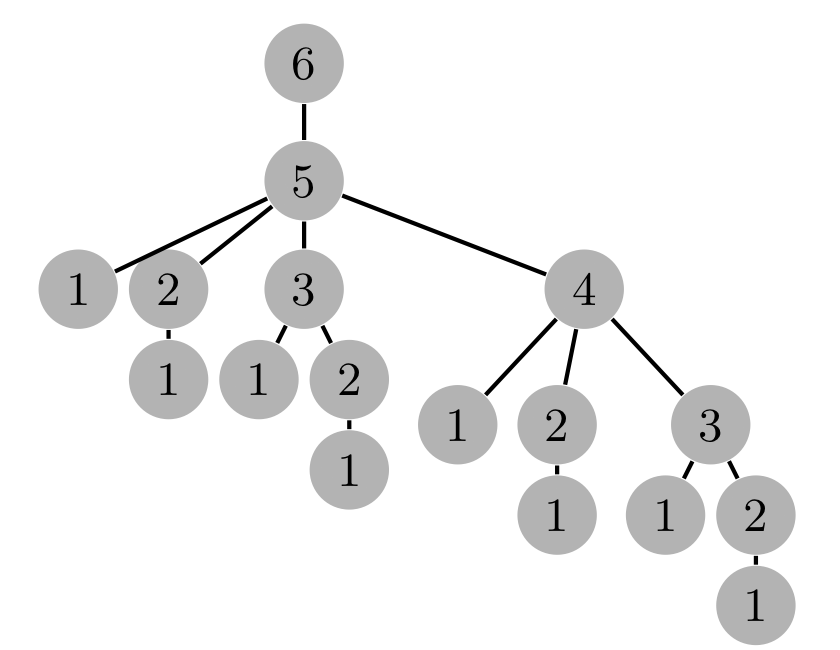

Supplement: Figure S1 — Tree . Tree with labels, for . (TIF) [file pone.0041900.s001.tif]
