# Supplementary material for: Human Matching Behavior in Social Networks: An Algorithmic Perspective
Source: PLoS One. 2012 Aug 22;7(8):e41900. doi: 10.1371/journal.pone.0041900 (PMC3425504; doi:10.1371/journal.pone.0041900)
Supplement: Text S1 — Technical proofs. This documents contains the detailed proofs of all technical results presented in the article. (PDF) [file pone.0041900.s002.pdf]

Human matching behavior in social networks: an algorithmic perspective – Proofs of the Theorems

Lorenzo Coviello[1,*], Massimo Franceschetti[1], Mathew McCubbins[2], Ramamohan Paturi[3], Andrea Vattani[3]
1 Department of Electrical and Computer Engineering, University of California San Diego, CA
2 Department of Political Science, University of Southern California, Los Angeles, CA
3 Department of Computer Science and Engineering, University of California San Diego, CA
∗ **E-mail: lcoviell@ucsd.edu**

Proof of Theorem 1.

For ease of presentation, we assume $p = 0$, and remark that this result holds for all choices of $0 \le p < 1$. Let G be a graph of n nodes and maximum degree Δ. Let m be the number of matched nodes in the smallest maximal matching of G. For $t \ge 0$, denote by W_t the set of nodes of G which are unmatched and have at least an unmatched neighbor at the beginning of round t, and let $|W_t|$ be the cardinality of W_t. Also, let M_t be the matching of G obtained by the Prudence algorithm at the beginning of round t and N_t be the number of nodes matched by M_t. For $t \ge 0$, define the random variable

$$D_t = m - N_t.$$

We devote the rest of the proof to showing that

$$E[D_t] \le (1 - (\Delta + 1)^{-3})^t E[D_0] \tag{1}$$

The theorem then follows by the observations that $E[D_0] \le n$ and that any maximal matching is at least a 1/2-approximation of the maximum matching.

To prove (1), we will first show that $E[B_t(W_t)|W_t] \ge (\Delta + 1)^{-3}|W_t|$, where $B_t(W_t)$ is the number of nodes in W_t that match with nodes in W_t during round t (here the expectation is taken over the randomness of the algorithm during round t). For $u \in W_t$, let $Z_t(u)$ be the indicator random variable that takes value 1 if and only if u gets matched with a node in W_t during round t. By linearity of expectation, we have that

$$E[B_t(W_t)|W_t] = \sum_{u \in W_t} E[Z_t(u)] = \sum_{u \in W_t} \Pr(Z_t(u) = 1).$$

Let A_t be the set of nodes $u \in W_t$ such that (i) u has no incoming or outgoing request to nodes in W_t, and (ii) all neighbors $v \in W_t$ of u have an incoming request. Let $\bar{A}_t = W_t \setminus A_t$. For $u \in A_t$, we have that $\Pr(Z_t(u) = 1) = 0$, as unmatched nodes prefer neighbors who requested them over other unmatched neighbors. On the other hand, for $u \in \bar{A}_t$, we have $\Pr(Z_t(u) = 1) \ge \Delta^{-2}$. To see this, note that a pending request involving u (if any) will be honored with probability at least Δ^{-2}; if no such request exists, the co-occurrence of the event of u requesting a neighbor with no incoming request and of that neighbor requesting u happens with probability at least Δ^{-2}. By definition of A_t, no two nodes in A_t can be neighbors. Also, by definition of W_t, every node $u \in W_t$ has at least one neighbor in W_t. These two facts imply that $|\bar{A}_t| \ge (\Delta + 1)^{-1} W_t$. We can conclude that $E[B_t(W_t)|W_t] \ge (\Delta + 1)^{-3}|W_t|$.

We now relate D_{t+1} to $B_t(W_t)$. First, note that $D_{t+1} \le D_t - B_t(W_t)$. By itself, this bound is not strong as W_t can be small. However, when W_t is small, the current matching must be close to a maximal matching. Indeed, by considering the union of M_t and any maximal matching of W_t, we have that

$m \leq N_t + |W_t|$. This implies that $D_t = m - N_t \leq |W_t|$ and hence $D_{t+1} \leq D_t - B_t(W_t) \leq |W_t| - B_t(W_t)$. Therefore, we have

$$D_{t+1} \leq D_t - B_t(W_t),$$
$$D_{t+1} \leq |W_t| - B_t(W_t).$$

By taking the expectations with respect to the randomness of the algorithm during round t, we get

$$E[D_{t+1}|W_t, D_t] \leq D_t - E[B_t(W_t)|W_t] \leq D_t - (\Delta+1)^{-3}|W_t|,$$
$$E[D_{t+1}|W_t, D_t] \leq |W_t| - E[B_t(W_t)|W_t] \leq |W_t| - (\Delta+1)^{-3}|W_t| = (1-(\Delta+1)^{-3})|W_t|.$$

Now, by taking the expectation with respect to the randomness of the algorithm during rounds up to t, we obtain

$$E[D_{t+1}] \leq E[D_t] - (\Delta+1)^{-3}E[|W_t|],$$
$$E[D_{t+1}] \leq (1-(\Delta+1)^{-3})E[|W_t|].$$

Letting $d_t = E[D_t]$, $w_t = E[|W_t|]$, and $\alpha = (\Delta+1)^{-3}$, the bounds above can be rewritten as

$$d_{t+1} \leq \min\left\{d_t - \alpha w_t, (1-\alpha)w_t\right\}.$$

To conclude the proof of (1), we show by induction that $d_t \leq d_0(1-\alpha)^t$. For $t = 0$, as $d_0 \leq w_0$, we have $d_1 \leq d_0 - \alpha w_0 \leq (1-\alpha)w_0$. Now, let us consider any $t \geq 1$ and distinguish between the cases of $w_t \leq d_0(1-\alpha)^t$ and $w_t > d_0(1-\alpha)^t$. If $w_t \leq d_0(1-\alpha)^t$, we have $d_{t+1} \leq (1-\alpha)w_t \leq d_0(1-\alpha)^{t+1}$. Otherwise, if $w_t > d_0(1-\alpha)^t$, using the induction hypothesis, we have that

$$d_{t+1} \leq d_t - \alpha w_t \leq d_0(1-\alpha)^t - \alpha w_t \leq d_0(1-\alpha)^t - d_0\alpha(1-\alpha)^t = d_0(1-\alpha)^{t+1},$$

which completes the proof.

Proof of Theorem 2.

For ease of presentation, we assume $p = 0$, and remark that this result holds for all choices of $0 \leq p < 1$. Let G be a graph of n nodes, maximum degree Δ, and maximum matching of size OPT. We will consider the unmatched nodes as *particles* randomly moving on the nodes of the network as per the algorithm choices. To see how the particle move, consider the particle positioned at any unmatched node u. If u requests a matched neighbor v and v accepts the requests, then the particle will move to v's old partner (which is left unmatched). If u requests an unmatched neighbor z and z accepts the request, then both the particles at u and z will dissolve. Note that when two particles dissolve the size of the matching increases by one.

An augmenting path is a path of odd length which alternates matched and unmatched edges and whose extreme edges are unmatched. Observe that by switching each unmatched edge of an augmenting path into a matched edge, and viceversa, the size of the matching increases by one.

We split the rounds into epochs of $\lfloor 1/\epsilon \rfloor$ rounds each. We claim that if at the beginning of any epoch the size of the matching is less than a $(1-\epsilon)$OPT, then the size of the matching increases by at least one by the end of that epoch with probability at least $\Delta^{-2/\epsilon}$. To prove the claim, consider the first round of any epoch and let $u_0, u_1, \ldots, u_\ell$ be any *shortest* augmenting path at the beginning of that round. It must be that $\ell < 2(\epsilon^{-1} - 1)$, otherwise Lemma 1 would imply that the size of the matching is at least a $\frac{\ell+1}{\ell+3} \geq 1 - \epsilon$ fraction of OPT. For $\ell = 1$, u_0 and u_1 will match with each other during the first round with probability at least Δ^{-2}, hence the claim is true. For $\ell = 3$, u_0 and u_3 will request respectively

u_1 and u_2 with probability at least Δ^{-2} during the first round of the epoch, and these requests will be accepted in the second round with probability at least Δ^{-2} — hence, the size of the matching increases by one within 2 rounds with probability at least Δ^{-4}. Now consider $5 \le \ell < 2(\epsilon^{-1} - 1)$. We have that two particles occupy the nodes u_0 and u_ℓ at the extremes of the augmenting path. With probability at least Δ^{-2}, u_0 requests to match with u_1 during the first round and u_1 accepts in the second round, making the corresponding particle move from u_0 to u_2. A similar argument yields that the particle at u_ℓ moves to $u_{\ell-2}$ within two rounds with probability at least Δ^{-2}. Moreover, as the augmenting path under consideration is a shortest augmenting path, nodes $u_2, \ldots, u_{\ell-2}$ have no unmatched neighbors at the beginning of the first round and hence do not receive any matching request during that round. Therefore, with probability at least Δ^{-4}, at the end of the second round the nodes u_2 and $u_{\ell-2}$ are unmatched whereas nodes $u_3, \ldots, u_{\ell-3}$ did not change their partner. That is, the length of the shortest path at the beginning of the third round of the epoch is at most $\ell - 4$ with probability at least Δ^{-4}. By means of the same argument, we can conclude that with probability at least $(\Delta^{-4})^{\ell/4} > \Delta^{-2/\epsilon}$, all nodes in an augmenting path are matched within $\ell/2 \le \lfloor 1/\epsilon \rfloor$ rounds, which proves the claim.

For any epoch i, we now associate a binary random variable X_i which takes on value 1 with probability $p = \Delta^{-2/\epsilon}$. The claim guarantees that the size of the matching after T epochs is at least $\min\{(1-\epsilon)\mathrm{OPT}, \sum_{i=1}^{T} X_i\}$. Also, as successive rounds of the algorithm are independent, the X_i's are independent random variables. For any $0 < \delta \le 1$, the Chernoff bound states that

$$\Pr\left[\sum_{i=1}^{T} X_i < (1-\delta)Tp\right] < \exp(-Tp\delta^2/2).$$

For any $c \ge 1/2$, by setting $T := cn\Delta^{-2/\epsilon}$ and $\delta := \epsilon$, the above yields that the size of the matching after T epochs (i.e., after $T\lfloor 1/\epsilon \rfloor \le \frac{c}{\epsilon} n \Delta^{2/\epsilon}$ rounds) is at least $\min\{(1-\epsilon)\mathrm{OPT}, (1-\epsilon)cn\} = (1-\epsilon)\mathrm{OPT}$ with probability at least $1 - \exp(-c\epsilon^2 n/2)$.

Proof Theorem 3.

Analysis.

For ease of presentation, we assume $p = 0$, and remark that this result holds for all choices of $0 \le p < 1$. We say that the nodes $\{a_i : 1 \le i \le n\} \cup \{b_i : 1 \le i \le n\}$ constitute the upper half of G_n, and the remaining ones constitute the lower half of G_n. Let $\mathcal{M} = \mathcal{M}_1 \cup \mathcal{M}_2$ be the set of all matchings of G_n of size $2n-1$, where $\mathcal{M}_1$ is the set of matchings of size $2n-1$ in which the two unmatched nodes are in opposite halves of G_n, and $\mathcal{M}_2 = \mathcal{M} \setminus \mathcal{M}_1$ are the remaining ones.

Our goal is to show that the PRUDENCE algorithm requires $2^{\Omega(n/\log^2 n)}$ rounds with high probability to reach the perfect matching of G_n when starting from any matching in $\mathcal{M}_1$. We first prove certain properties for the matchings in $\mathcal{M}_1$. We then establish a correspondence between the Markov chain over matchings induced by the PRUDENCE algorithm and a classical random walk on the tree T_n^*. In particular, we show that the hitting time of the root of T_n^* is a lower bound on the number of rounds to reach the perfect matching of G_n.

Properties of matchings in $\mathcal{M}_1$.

We begin by characterizing the matchings in $\mathcal{M}_1$.

Lemma 1 *Consider any matching $M \in \mathcal{M}_1$, and let a_k, b_ℓ be the unmatched nodes in the upper and lower half of G_n, respectively. Then, the following properties hold:*

1. *For all $i < k$ and $i > \ell$, the matching M contains the edges (a_i, b_i).*

2. *If $k < n$, M contains the edge (a_n, b_j) for some $1 \leq j < n$. Similarly, if $\ell > n+1$, M contains the edge (a_i, b_{n+1}) for some $n+1 < i \leq 2n$. That is, the nodes a_n and b_{n+1} can be matched only through non-horizontal edges.*

3. *If in its upper half M contains a pair of edges $(a_{i_1}, b_{j_1}), (a_{i_2}, b_{j_2})$ with $i_1 \neq j_1$, $i_2 \neq j_2$, and $1 \leq i_1 < i_2 \leq n$, then $1 \leq k \leq j_1 < i_1 \leq j_2 < i_2 \leq n$. Similarly, if in its lower half M contains a pair of edges $(a_{i_1}, b_{j_1}), (a_{i_2}, b_{j_2})$ with $i_1 \neq j_1$, $i_2 \neq j_2$, and $n+1 \leq j_1 < j_2 \leq 2n$, then $n+1 \leq j_1 < i_1 \leq j_2 < i_2 \leq \ell \leq 2n$. That is, non-horizontal matching edges do not cross.*

Proof. To prove the first property, we show that $(a_i, b_i) \in M$ for all $i < k$ (the claim for $i > \ell$ can be proven in the same way). We show by induction on $1 \leq j \leq k-1$ that $(a_i, b_i) \in M$ for all $i \leq j$. For $j = 1$, we have that a_1 must be matched to b_1 (its only neighbor), and therefore the claim holds true. Suppose the claim holds true for some $j < k-1$. By the inductive assumption we have that $(a_i, b_i) \in M$ for all $i \leq j$. As $(a_{j+1}, b_i) \in E$ if and only if $i \leq j+1$, a_{j+1} must be matched to b_{j+1}, and therefore the claim holds for $j+1$.

The second property follows by observing that $M \in \mathcal{M}_1$ implies that the bridge edge (a_{n+1}, b_n) is in M, and therefore a_n cannot be matched to b_n, and a_{n+1} cannot be matched to b_{n+1} in M. To see this, suppose by contradiction that $(a_{n+1}, b_n) \notin M$. Then, b_n must be matched to a_n (its only neighbor besides a_{n+1}), and a node in $\{a_1, \ldots, a_{n-1}\}$ is unmatched. Then, each of the $n-1$ nodes in $\{b_1, \ldots, b_{n+1}\}$ must be matched with one of the $n-2$ matched nodes in $\{a_1, \ldots, a_{n-1}\}$, generating a contradiction. This implies that $(a_{n+1}, b_n) \in M$.

To prove the third property, assume that, in its upper half, M contains edges $(a_{i_1}, b_{j_1}), (a_{i_2}, b_{j_2})$ with $i_1 \neq j_1$, $i_2 \neq j_2$, and $1 \leq i_1 < i_2 \leq n$. Then, it must be that $j_1 < i_1$ and $j_2 < i_2$. Moreover, Property 1 implies that $k \leq j_1$. Therefore, it only remains to show that $i_1 \leq j_2$. Suppose by contradiction that $i_1 > j_2$. As $i_1 > j_1 \geq k$ and $j_1 \neq j_2$, it must be that $i_1 \geq k+2$. As b_{j_2} is matched to a_{i_2} and $i_2 > i_1$, we have that each of the $i_1 - k \geq 2$ nodes in $\{a_{k+1}, \ldots, a_{i_1}\}$ must be matched to one of the $i_1 - k - 1$ nodes in $\{b_k, \ldots, b_{i_1 - 1}\} \backslash \{b_{j_2}\}$, generating a contradiction. This implies that $i_1 \leq j_2$ and therefore $1 \leq k \leq j_1 < i_1 \leq j_2 < i_2 \leq n$. The claim in Property 3 regarding the lower half of M is similarly proved. ∎

It follows from Lemma 1 that a matching $M \in \mathcal{M}_1$ can be uniquely reconstructed by specifying the two unmatched nodes and the nodes in $\{a_1, \ldots, a_n\} \cup \{b_{n+1}, \ldots, b_{2n}\}$ whose matching edges are *non-horizontal.* To see this, consider the upper half of G_n: assume $a_{j_0} \neq a_n$ is the unmatched node and $S = \{j_1, \ldots, j_m\}$, with $1 \leq j_0 < j_1 < j_2 < \ldots < j_m = n$, is the set of indexes of the left nodes whose matching edges are non-horizontal. (Note that $n \in S$ by Lemma 1.) Then, $j_0 < j_1$ and $(a_i, b_i) \in M$ for all i such that $i \notin S \cup \{j_0\}$ and $1 \leq i \leq n$. Hence, it necessarily holds that $(a_{j_i}, b_{j_{i-1}}) \in M$ for all $1 \leq i \leq m$. This completes the construction of the matching in the upper half of G_n. A similar argument can be applied to the lower half. These two arguments imply the following lemma.

Lemma 2 *There exists a bijection ψ between matchings in $\mathcal{M}_1$ and elements of $\mathcal{P} \times \mathcal{P}'$, where*

$$\mathcal{P} = \big\{(x, S) : x \in \{1, \ldots, n-1\}, \{n\} \subseteq S \subseteq \{x+1, \ldots, n\}\big\} \cup \{(n, \emptyset)\},$$
$$\mathcal{P}' = \big\{(y, S') : y \in \{n+1, \ldots, 2n\}, \{n+1\} \subseteq S' \subseteq \{n+1, \ldots, y-1\}\big\} \cup \{(n+1, \emptyset)\}.$$

The tree T_n^*.

Definition 1 *Let T_1 be a labelled rooted tree with a singleton node with label 1. Inductively, for $2 \leq i \leq n-1$, let T_i be the labelled rooted tree whose root is labelled with i and its children are $T_1, \ldots, T_{i-1}$. We define T_n^* to be the tree with an unlabelled root whose only child is T_n (also see Figure S1). Let r^* denote the root of T_n^*.*

We show that the hitting time of r^* when starting at any node $u \neq r^*$ is exponential with high probability. For a node $\neq r^*$, we call the edge that connects u to its parent u's *exit* edge. For any subtree $T_i \subset T_n^*$, let Z_i be the random variable denoting the number of steps that it takes for a walk starting at the root of T_i to "exit" T_i. The following lemma provides an exponential lower bound on Z_i

Lemma 3 *There exist positive constants $\alpha, \gamma > 0$ such that, for all $i \geq 2$,*

$$\Pr[Z_i \geq \gamma \cdot 2^{i/(\alpha \log^2 i)}] \geq 1 - \frac{1}{\log i}.$$

Proof. We proceed by induction on i. For convenience, define $g(i) = \alpha \log^2 i$ and $f(i) = \gamma \cdot 2^{i/g(i)}$ for some $\alpha, \gamma > 0$. For any constant $\alpha > 0$, there exists a small enough constant $\gamma > 0$ such that $f(i) \leq 1$; therefore, as $Z_i \geq 1$ with probability 1, the claim holds trivially for any $i \leq i^*$, where i^* is a suitable large constant.

Now consider any $i \geq i^*$ and suppose the claim holds up to $i-1$. Every time the walk is on the root of T_i, it exits T_i with probability $1/i$. Therefore, letting E_t be the event that the first t times the walk is on the root of T_i it does *not* exit T_i, we have $\Pr[E_t] \geq 1 - t/i$. Let $t = i/(2\log i)$, and let D_j, $1 \leq j \leq t$, be the event that, when it is on the root of T_i for the j-th time, the walk moves to the root of one of the subtrees $T_{i-g(i)}, \ldots, T_{i-1}$ *and* takes at least $f(i - g(i))$ steps to exit that subtree. For $1 \leq j \leq t$, we have

$$\begin{aligned}\Pr[D_j \mid E_t] &\geq \frac{g(i)}{i} \cdot \Pr[Z_{i-g(i)} \geq f(i-g(i))] \\ &\geq \frac{g(i)}{i} \cdot \left(1 - \frac{1}{\log(i-g(i))}\right),\end{aligned}$$

by the induction hypothesis on $Z_{i-g(i)}$. Letting χ_j be the indicator function of the event D_j for $1 \leq j \leq t$, the probability that at least two of the events D_j happen, given E_t, is lower bounded by:

$$\Pr\left[\sum_{j=1}^{t} \chi_j \geq 2 \,\middle|\, E_t\right] \geq \Pr\left[\sum_{j=1}^{t/2} \chi_j \geq 1, \sum_{j=t/2+1}^{t} \chi_j \geq 1 \,\middle|\, E_t\right] = \Pr\left[\sum_{j=1}^{t/2} \chi_j \geq 1 \,\middle|\, E_t\right]^2.$$

By union bound, we can write

$$\begin{aligned}\Pr\left[\sum_{j=1}^{t/2} \chi_j \geq 1 \,\middle|\, E_t\right] &\geq 1 - \prod_{i=1}^{t/2} (1 - \Pr[D_j | E_t]) \\ &\geq 1 - \left(1 - \frac{g(i)}{i}\left(1 - \frac{1}{\log(i-g(i))}\right)\right)^{t/2} \\ &\geq 1 - \exp\left[-\frac{\alpha \log i}{4}\left(1 - \frac{1}{\log(i-g(i))}\right)\right] \\ &\geq 1 - \frac{1}{i^{\alpha/8}},\end{aligned}$$

where the last step holds for i sufficiently large so that $\log(i - g(i)) \geq 2$. This implies that

$$\Pr\left[\sum_{j=1}^{t} \chi_j \geq 2 \,\middle|\, E_t\right] \geq \left(1 - \frac{1}{i^{\alpha/8}}\right)^2 \geq 1 - \frac{2}{i^{\alpha/8}}.$$

Therefore, we conclude that

$$\Pr[Z_i \geq 2 \cdot f(i-g(i))] \geq \Pr\left[\sum_{j=1}^{t} \chi_j \geq 2\right] \geq \Pr\left[\sum_{j=1}^{t} \chi_j \geq 2 \,\middle|\, E_t\right] \Pr[E_t] \geq \left(1 - \frac{2}{i^{\alpha/8}}\right)\left(1 - \frac{t}{i}\right) \geq 1 - \frac{1}{\log i},$$

where the last step holds by choosing α sufficiently large. The claim now follows since $2 \cdot f(i-g(i)) \geq f(i)$. ∎

Note that any random walk starting at any node $u \neq r^*$ has to exit T_n before hitting r^*. Therefore, an application of Lemma 3 to T_n yields a lower bound to the hitting time of r^* when starting at any node $u \neq r^*$.

Corollary 1 *The hitting time of r^* of a random walk starting at any node $u \neq r^*$ is $2^{\Omega(n/\log^2 n)}$ with high probability.*

Proof of of Theorem 3.

Figure 1. The bad graph. The "bad" graph G_n for $n = 3$. One of the "bad" matchings of Theorem 3 is highlighted in red.

For $t \geq 0$, let $\mathcal{M}(t)$ be the matching at the beginning of round t and assume $\mathcal{M}(0) \in \mathcal{M}_1$. To analyze the convergence to a perfect matching, we will consider on the event that $\mathcal{M}(t) \notin \mathcal{M}_1$. Note that in order for this event to happen, the bridge edge (a_{n+1}, b_n) of G_n will have to swap out of the matching. Let $E(t)$ be the event that a_n requests b_n during round t. Similarly, let $E'(t)$ be the event that b_{n+1} requests a_{n+1} during round t. Define the random variables

$$\begin{aligned}\tau_n &= \min\{t : E(t) \text{ happens}\},\\ \tau_n' &= \min\{t : E'(t) \text{ happens}\},\\ \tau_n^* &= \min\{\tau_n, \tau_n'\}.\end{aligned}$$

Then τ_n^* is a lower bound on the number of rounds to reach the perfect matching. Lemma 4 below states that, for some $c > 0$,

$$\Pr\left[\tau_n \leq 2^{cn/\log^2 n} \;\middle|\; \tau_n \leq \tau_n'\right] = o(1) \qquad \text{and} \qquad \Pr\left[\tau_n' \leq 2^{cn/\log^2 n} \;\middle|\; \tau_n' \leq \tau_n\right] = o(1).$$

Then the main theorem follows as

$$\begin{aligned}\Pr\left[\tau_n^* \leq 2^{cn/\log^2 n}\right] &= \Pr\left[\tau_n^* \leq 2^{cn/\log^2 n} \;\middle|\; \tau_n \leq \tau_n'\right] \Pr\left[\tau_n \leq \tau_n'\right] + \Pr\left[\tau_n^* \leq 2^{cn/\log^2 n} \;\middle|\; \tau_n' < \tau_n\right] \Pr\left[\tau_n' < \tau_n\right]\\ &= \Pr\left[\tau_n \leq 2^{cn/\log^2 n} \;\middle|\; \tau_n \leq \tau_n'\right] \Pr\left[\tau_n \leq \tau_n'\right] + \Pr\left[\tau_n' \leq 2^{cn/\log^2 n} \;\middle|\; \tau_n' < \tau_n\right] \Pr\left[\tau_n' < \tau_n\right]\\ &= o(1).\end{aligned}$$

Lemma 4

$$\Pr\left[\tau_n \leq 2^{cn/\log^2 n} \;\middle|\; \tau_n \leq \tau_n'\right] = o(1) \qquad \textit{and} \qquad \Pr\left[\tau_n' \leq 2^{cn/\log^2 n} \;\middle|\; \tau_n' \leq \tau_n\right] = o(1).$$

Proof. We will prove the first bound. The second one follows by symmetry. Conditioning on the event that $\tau_n \leq \tau_n'$, we will analyze the matching in the upper half of G_n induced by $\mathcal{M}(t)$. Since $\tau_n \leq \tau_n'$, $\mathcal{M}(t) \in \mathcal{M}_1$ as long as $E(t)$ does not happen. By Lemma 2, it is equivalent to study the Markov process $\{(X(t), \mathcal{S}(t)), t \geq 0\}$ over $\mathcal{P} \cup \{(\perp, \emptyset)\}$, where $(X(t), \mathcal{S}(t))$ is defined as the first marginal of $\psi(\mathcal{M}(t))$, and the additional state $(\perp, \emptyset)$ is reached when the event $E(t)$ happens. That is, conditioning on the event $\tau_n \leq \tau_n'$, it follows that

$$\tau_n = \min\{t : (X(t), \mathcal{S}(t)) = (\perp, \emptyset)\}. \tag{2}$$

If $\tau_n \leq \tau'_n$ and $(X(t), \mathcal{S}(t)) \neq (\perp, \emptyset)$, all the neighbors of the unmatched node in the upper half of G_n are matched at the beginning of round t, and hence are *equally likely* to be requested during round t. Therefore, the Markov process $(X(t), \mathcal{S}(t))$ has the following transition probabilities.

$$\Pr\Big[(X(t+1), \mathcal{S}(t+1)) = (x', S') \ \Big| \ (X(t), \mathcal{S}(t)) = (x, S) \neq (\perp, \emptyset), \tau_n \leq \tau'_n\Big] = \frac{1}{x},$$

for any

$$(x', S') \in \begin{cases} \big\{(x'', S \cup x) : x'' < x\big\} \cup \big\{(\min(S), S \setminus \min(S))\big\}, & \text{if } x < n \text{ (and } S \neq \emptyset) \\ \big\{(x'', S \cup x) : x'' < x\big\} \cup \big\{(\perp, \emptyset)\big\}, & \text{if } x = n \text{ (and } S = \emptyset) \end{cases}$$

The case $(x', S') \in \{(x'', S \cup x) : x'' < x\}$ represents the scenario in which the unmatched node a_x requests a node through a non-horizontal edge: in this case, no progress is made as the unmatched node in the next round will be further away from a_n. If the unmatched node a_x requests the node on its horizontal edge, the next unmatched node will be closer to a_n. In the special case $(x, S) = (n, \emptyset)$, if the unmatched node requests the neighbor on its horizontal edge, then the bridge edge is swapped out of the matching and $\mathcal{M}(t+1) \notin \mathcal{M}_1$.

We will now show that the Markov chain $\{(X(t), \mathcal{S}(t)), t \geq 0\}$ is equivalent to the random walk on T_n^*. For a node v of T_n^*, let x_v be its label and S_v be the set of labels of its ancestors. Define the function ϕ from nodes of T_n^* to states of the chain as follows:

$$\phi(v) = \begin{cases} (\perp, \emptyset), & v = r^* \\ (x_v, S_v), & v \neq r^* \end{cases}$$

It is easy to verify that ϕ is a bijection. Two nodes u and v are adjacent in T_n^* if and only if there is a nonzero transition probability between the states $\phi(u)$ and $\phi(v)$. To see this, suppose there is a nonzero transition probability from (x_u, S_u) to (x_v, S_v) in the Markov chain. Let $u = \phi^{-1}(x_u, S_u)$ and $v = \phi^{-1}(x_v, S_v)$ be the corresponding nodes in T_n^*. There are two cases: (a) if $x_v < x_u$ then $S_v = S_u \cup x_u$, and v is a child of u; (b) if $x_v > x_u$ then $x_v = \min(S_u)$, $S_v = S_u \setminus \min(S_u)$, and v is the parent of u. The other direction is analogous. Therefore, conditioning on $\tau_n \leq \tau'_n$ and $(X(0), \mathcal{S}(0)) \neq (\perp, \emptyset)$, we can conclude that $\min\{t : (X(t), \mathcal{S}(t)) = (\perp, \emptyset)\}$ equals the hitting time of r^* for a random walk on T_n^* starting at the node $\phi^{-1}(X(0), \mathcal{S}(0)) \neq r^*$. The lemma follows by equation (2) and Corollary 1. ∎
